# Effects of Florfenicol on Intestinal Structure, Microbial Community and Antibiotic Resistance Genes in *Penaeus vannamei*

**DOI:** 10.3390/microorganisms14010204

**Published:** 2026-01-15

**Authors:** Gengshen Wang, Xinyong Shi, Yi Yan, Jianjun Xie, Demin Zhang, Huajun Zhang

**Affiliations:** 1 State Key Laboratory for Managing Biotic and Chemical Threats to the Quality and Safety of Agro-Products, Ningbo University, Ningbo 315211, China; wanggs@zjou.edu.cn (G.W.); huynhngocchau41412@gmail.com (X.S.); kershrintal8efiud@hotmail.com (Y.Y.); zhangdemin@nbu.edu.cn (D.Z.); 2 Ministry of Education Key Laboratory of Applied Marine Biotechnology, School of Marine Sciences, Ningbo University, Ningbo 315211, China; 3 Zhejiang Marine Fisheries Research Institute, Zhoushan 316021, China

**Keywords:** florfenicol, *Penaeus vannamei*, histopathology, microbial community, pathogenic bacteria, antibiotic resistance genes

## Abstract

Antibiotic feeding in shrimp farming is an optional practice conducted with the aim of preventing and controlling bacterial diseases. However, the administration of antibiotics can disrupt the microbiota of both shrimp and surrounding environment, potentially compromising host health. Given the limited effective antibiotic options in aquaculture, it is crucial to evaluate the effects of florfenicol (FF) on the intestinal health of shrimp and the associated microbial communities. This study first investigated the impact of FF on the intestinal structure of *Penaeus vannamei* over two feeding durations (5 and 10 days), each followed by a 10-day basal diet recovery period. Simultaneously, variations in microbial communities and antibiotic resistance genes (ARGs) in both the intestine and rearing water were explored. The results showed that intestinal damage was aggravated with the extension of FF duration and gradually recovered after FF withdrawal. Significant changes in microbial composition and β-diversity were observed in both the rearing water and intestine following FF feeding. Extending the FF treatment to 10 days led to a reduced abundance of *Rhodobacteraceae* and an increased abundance of *Flavobacteriaceae* and *Vibrionaceae* in the intestine after 10 days of feeding the basic diet, which may pose a potential risk to shrimp health. Based on correlation analysis of ARGs, microbial communities and pathogenic bacteria, we speculated that rearing water may serve as a reservoir for ARGs dissemination compared to the shrimp intestine. These findings are of great importance for assessing the impact of administration duration under the FF therapeutic dose and highlight the potential risks associated with its overuse in shrimp farming.

## 1. Introduction

With the development of intensive aquaculture practices, *Penaeus vannamei* has become the most productive farmed crustacean species worldwide. However, shrimp aquaculture is increasingly challenged by infectious diseases, particularly those caused by bacterial pathogens such as *Vibrio*, *Pseudomonas* and *Aeromonas* [1,2,3]. As a third-generation amidoalcohol antibiotic, florfenicol (FF) exhibits potent antibacterial activity, broad-spectrum and relatively low side effects, making it one of the most applied chemotherapeutic agents for the control of one of the most applied chemotherapeutic agents for the control of shrimp pathogens [4,5]. Previous studies have preliminarily focused on the pharmacokinetics, tissue distribution and metabolic processes of FF in shrimp [6]. Nevertheless, its potential toxicological effects remain insufficiently understood. Accumulating evidence suggests that FF exposure can induce biomolecular damage and impair antioxidant defense systems [7]. Moreover, excessive use of FF may lead to hematopoietic suppression in aquatic organisms, accumulation of drug residues and the propagation of antibiotic resistance genes (ARGs) [8,9].

As the principal organ responsible for nutrient absorption and immune defense, the gastrointestinal tract plays a pivotal role in maintaining the growth and health of aquatic animals. It is also a primary target organ directly exposed to orally administered antibiotics. Recent studies have demonstrated that environmentally relevant concentrations of sulfamethoxazole can induce intestinal epithelial damage in grass carp (*Ctenopharyngodon idella*) [10]. In *Carassius auratus gibelio*, prolonged exposure to sulfadiazine resulted in significant intestinal pathological changes, including microvilli exfoliation, submucosa thickening, vacuolar degeneration and muscularis necrosis [11]. Similarly, FF exposure in aquatic crustaceans has been reported to cause markedly shortening of intestinal villi and thinning of the muscle layers in *Eriocheir sinensis* [12]. Despite these findings, information regarding the effects of FF feeding, particularly over extended durations, on the intestinal structure of shrimp remains limited.

Gastrointestinal microbiota are essential for aquatic animal health, contributing to nutrition, epithelial development, immunity and pathogen resistance [13,14]. Dysbiosis of the intestinal microbial community may facilitate pathogen proliferation and ultimately impair host health [15]. Current studies have demonstrated that antibiotic exposure can substantially alter the composition and diversity of intestinal microbiota, thereby destabilizing its equilibrium [16,17]. For instance, a standard therapeutic 10-day treatment with FF significantly reshaped the structure of the intestinal microbiota and reduced bacterial diversity in channel catfish [18]. Moreover, FF feeding may enhance the relative abundance of potentially pathogenic gut microorganisms, with more pronounced effects observed following prolonged exposure [19]. In *Apostichopus japonicus*, continuous FF feeding disrupted microbial ecological networks by reducing the abundance of key taxa, leading to intestinal microbiota dysbiosis [20].

In addition to host-associated microbiota, microorganisms in rearing water are increasingly recognized as critical determinants of shrimp health [21], as they can regulate water quality and influence shrimp growth and disease resistance. Aquatic animals are in constant contact with the surrounding water, and changes in the rearing water microbiome can directly shape the intestinal microbiota through water ingestion [22,23]. Because antibiotics cannot be effectively metabolized by aquatic animals, biologically active antibiotic residues are often released into the rearing environment [24], leading to alterations in the structure and diversity of bacterial communities in rearing water [25]. However, the succession of rearing water microbiota following FF feeding in shrimp aquaculture systems remains poorly characterized.

The persistent use of antibiotics is widely recognized as a major driver of antibiotic resistance [26] and may promote the enrichment and dissemination of ARGs in the aquaculture environment [27]. Feeding Pacific white shrimp with diets supplemented with ciprofloxacin and sulfonamide has been shown to significantly increase the abundance of ARGs in the intestinal microbiota [28]. Similarly, the therapeutic level of FF treatment also notably promoted the proliferation of ARGs in channel catfish tanks [18]. Oral administration of FF has been reported to increase the total relative abundance of ARGs and mobile genetic elements (MGEs) in the intestine of *Piaractus mesopotamicus*, potentially facilitating ARG enrichment and horizontal transfer within the aquaculture system [29]. However, systematic investigations into the temporal dynamics of ARGs in shrimp cultivation systems during and after FF exposure remain scarce, particularly from the perspective of practical farming scenarios.

The effects of antibiotics on commensal microbial communities are influenced by multiple factors, including dosage, treatment duration and the intrinsic composition and resistance profiles of the microbiota [30,31]. Several studies have demonstrated that different FF doses alter intestinal microbiota composition and diversity, accompanied by significant histological changes [12,32,33]. Notably, high-dose FF exposure has been shown to induce intestinal dysfunction and disrupt gut microbiota homeostasis in Chinese mitten crab [12]. However, the effects of FF treatment duration on the intestinal health of shrimp remain largely unexplored. In aquaculture practice, extending antibiotic treatment duration is often adopted to improve therapeutic efficacy. Therefore, the present study employed a tank-based shrimp cultivation system to systematically evaluate the effects of FF treatment duration on the intestinal morphology, microbial community structure and ARGs in shrimp. This work aims to provide a comprehensive evaluation of shrimp intestinal health in response to FF exposure duration and to elucidate its broader implications for aquaculture environment safety.

## 2. Materials and Methods

### 2.1. Experimental Design and Sample Collection

The experiment was conducted at the Xixuan Island Base of Zhejiang Marine Fisheries Research Institute in Zhoushan, Zhejiang Province, China (29.89° N, 122.31° E). A total of 800 specific pathogen-free shrimp (5.73 ± 0.42 g) were obtained from the base and acclimated for 7 days in ten tanks containing 400 L of sanitized seawater. Each tank was randomly stocked with 80 healthy individuals. The tanks were randomly assigned to two experimental groups, each with five replicates. Shrimp in group D were fed a FF-supplemented diet for 5 days, corresponding to the standard therapeutic duration, followed by a basal diet (Ningbo Tech-Bank Co., Ltd., Ningbo, China) for an additional 15 days. In contrast, Shrimp in group T received the FF-supplemented diet for 10 days and were subsequently fed the basal diet for the remaining 10 days. The FF-supplemented diet was prepared by adding FF at a standard therapeutic dose of 15 mg/kg shrimp body weight to the basal diet, in accordance with established recommendations [34]. FF powder (10% purity) for aquaculture use was commercially obtained from Nanhua Qianmu Biotechnology Co., Ltd. (Zhengzhou, Henan, China). Shrimp were fed three times daily at 6:30 am, 12:00 pm and 5:00 pm, with a feeding rate equivalent to 3% of the initial shrimp body weight. The feeding trial lasted for 20 days, during which approximately 10% of the rearing water was exchanged daily. Throughout the experimental period, water temperature ranged from 23.4 to 29.1 °C, salinity from 24.2 to 27.2‰, pH from 7.7 to 8.1, and dissolved oxygen levels were maintained above 5.0 mg/L.

At the beginning of the experiment, shrimp and rearing water were randomly collected from the ten tanks to serve as the original baseline samples (Control). Specifically, a total of 25 shrimp and corresponding rearing water samples were obtained, and five shrimp or rearing water samples were pooled to generate one composite biological sample per tank. Subsequently, five shrimp and their corresponding rearing water were sampled from each tank in groups D and T on days 5, 15 and 10, 20, respectively. For histopathological analysis, intestinal tissues (≥15 individuals per group) were collected and immediately soaked in Bourne’s fixative for 24 h, after which the samples were transferred to and stored in 70% ethanol until further processing. Microbial samples were collected from the intestines and stomachs of the shrimp, while rearing water samples were filtered through a 0.22 μm polycarbonate membrane (47 mm-diameter, Millipore, Billerica, MA, USA) to collect microbes. Muscle tissues were collected using sterile 5 mL centrifuge tubes for FF residue analysis. All samples were frozen and stored at −80 °C. In addition, 30 shrimp were randomly selected at the beginning and end of the experiment to measure individual body weight and length, which were used to calculate growth performance parameters.

The following parameters were used for evaluating the growth performance of shrimp:

$$\begin{array}{l} Survival\;rate\;(SR,\;\%)=100\%\times N_{t}/N_{0};\\ Specific\;growth\;rate\left(SRG,\;\%⁄day\right)=100\%\times \left(\ln W_{t}-\ln W_{0}\right)/t;\\ Length\;growth\;rate\left(LGR,\;\%⁄day\right)=100\%\times \left(\ln L_{t}-\ln L_{0}\right)/t; \end{array}$$

where *N_t_* and *N*_0_ are the final and initial shrimp quantities, respectively; *W_t_* and *W*_0_ represent the final and initial shrimp weights (g), respectively; *L_t_* and *L*_0_ represent the final and initial shrimp lengths (cm), respectively, in each tank; *t* is the trial period in days (day).

### 2.2. Histopathological Observation

The fixed intestinal tissues were trimmed to approximately 1–2 cm in both width and length and placed into histological cassettes. After a series of processes including ethanol dehydration, xylene transparency, and paraffin embedding, the tissues were serially sectioned at a thickness of 5 μm using a semi-automatic microtome Leica RM2016 (Leica Microsystems, Wetzlar, Germany). The sections were subsequently stained with hematoxylin–eosin (HE) and mounted with neutral resin. Histological structures were examined under a light microscope Leica ICC50W (Leica Microsystems, Wetzlar, Germany), and qualitative comparative analyses were conducted among experimental groups.

### 2.3. DNA Extraction and 16S rRNA Gene Amplicon Sequencing

Bacterial DNA was extracted from the intestine and stomach sample using a QIAamp DNA Stool Mini Kit (Qiagen, Hilden, Germany) and from the water sample using a Power Soil^®^ DNA isolation kit (MOBIO, Carlsbad, CA, USA) following the manufacturer’s instructions. DNA quality was assessed by electrophoresis on a 0.8% (*w*/*v*) agarose gel, and DNA concentration was quantified using a Nanodrop 2000 spectrophotometer (NanoDrop Technologies, Wilmington, DE, USA). The V3-V4 regions of the 16S rRNA gene were amplified using primers 341F (CCTAYGGGRBGCASCAG) and 806R (GGACTACNNGGGTATCTAAT). PCR products were detected with 0.8% agarose gel electrophoresis and purified using a QIAquick Gel Extraction Kit (Qiagen, Hilden, Germany). DNA Library construction was performed using the NEBNext^®^ Ultra™ II DNA Library Prep Kit (NEB, Ipswich, MA, USA) and quantified by Qubit and Q-PCR. After the library was qualified, Illumina’s NovaSeq platform PE250 (Illumina, San Diego, CA, USA) was used for sequencing. Comprehensive contamination controls were performed in microbiological analyses throughout the study.

### 2.4. Data Processing

Raw data was processed using Quantitative Insights into Microbial Ecology 2 (QIIME2, 2022.10). Quality control and chimaera removal of sequences were performed using fastp (v0.20.1) and vsearch (v2.14.0) software, and then DADA2 pipeline in QIIME2 was used for denoising to generate the amplicon sequence variants (ASVs). Representative sequences of each ASV were aligned against the SILVA 138 database to obtain taxonomic information [35]. To improve the quality of sequencing data, ASVs classified as chloroplasts, mitochondria and unclassified sequences were discarded. Due to insufficient sequencing depth, one sample was excluded. This step enhances the robustness of the retained data while acknowledging a potential limitation to the generalizability of the microbiome findings. Finally, the ASV feature table was rarefied to 12,386 sequences per sample to normalize the sequencing depth.

Representative sequences of each ASV were aligned and annotated with the database of pathogenic bacteria involved in water environment (DPiWE, dayuz.com, accessed on 11 December 2023) [36]. Potentially pathogenic ASVs were identified using a filtering threshold of an E-value < 1 × 10^−6^ and sequence identity > 97%. Due to difference among reference databases, the final annotation of sequences was based on the DPiWE database.

### 2.5. Quantification of ARGs

Real-time qPCR assays targeting the 16S rRNA gene, the integrase gene (*int*1) and four FF related ARGs (*flo*R, *cml*A, *cfr* and *fex*A) were conducted on the Bio-Rad CFX96 Touch system (Bio-Rad, Hercules, CA, USA) with AceQ qPCR SYBR Green Master Mix (Vazyme, Nanjing, China). The sequence of specific primers, annealing temperature and amplicon size were listed in Table 1. All qPCR assays were performed in 96-well plates with a final reaction volume of 15 μL, containing 7.5 μL of 2× SYBR Green Mix, 0.7 μL of each primer (10 μM), 1 μL of template DNA, and 5.1 μL ddH_2_O. Each qPCR run was conducted under the following conditions: initial denaturation at 95 °C for 5 min, 40 cycles of denaturation at 95 °C for 20 s, annealing temperature of target genes (Table 1) for 20 s, 72 °C for 40 s, and then melting curve analysis. Standard curves were generated using 10-fold serial dilutions of purified plasmid DNA containing the target gene inserts. The amplification efficiencies ranged from 94.02% to 103.94%, and the coefficients of determination (R^2^) for all standard curves exceeded 0.997, indicating high amplification efficiency and minimal PCR inhibition. The relative abundance of ARGs was calculated by normalizing ARG copy numbers to those of the 16S rRNA gene [27].

### 2.6. Determination of the Residual FF

The processes of shrimp muscle sample preparation and determination of FF concentration were carried out according to Chinese national standard GB/T 20756-2006 [40]. Briefly, 5 g of each homogenized sample was extracted twice with 15 mL of ethyl acetate. The combined extracts were evaporated to dryness at 40 °C, reconstituted in 2 mL of the initial mobile phase, and further purified using N-hexane. After filtration through a 0.22 μm polycarbonate membrane, the aqueous phase was injected into a vial and analyzed by liquid chromatography—tandem mass spectrometry (LC-MS-MS) using a 5 μm-particle C18 column (150 × 2.1 mm, 1.7 μm). The method detection limit for FF was 1 μg/kg, and the average recoveries in negative samples at concentrations ranging from 1 to 10 μg/kg were 87.1–102.1%.

### 2.7. Statistical Analysis

The α- and β-diversity indices were calculated by the R (v4.3.2) “vegan” package. Non-metric multidimensional scale (NMDS) analysis and analysis of similarities (ANOSIM) were applied to analyze the succession and similarity of microbial communities based on Bray–Curtis dissimilarity. Microbial community composition was visualized at the family level (taxa with an average relative abundance > 0.2% in at least one group), and differences in community composition among groups were assessed using Stamp (v2.1.3). SourceTracker analysis was conducted to identify the proportion of the shrimp intestine microbial community originating from rearing water and stomach [41]. Co-occurrence networks were constructed based on Spearman’s correlation matrices using the ‘Hmisc’ package (v5.2-5), in which robust correlations (|*ρ*| > 0.6, FDR-adjusted *p* value < 0.05) among ASVs were retained. Network properties were calculated and exported using the ‘igraph’ package (v2.2.1) according to previously described methods [42], and network visualization was performed using Cytoscape (v3.10.2) [43]. One-way analysis of variance (ANOVA) was applied to test for differences in shrimp growth parameters, bacterial α-diversity indices, and ARGs abundances among groups.

## 3. Results

### 3.1. Shrimp Growth and FF Concentration

After 20 days, the survival rate, specific growth rate and body length growth rate of shrimp in group D were slightly higher than those in group T, although these differences were not statistically significant (Table 2).

The concentration of residual FF in muscle in group D was lower than that in group T following FF feeding (*p* < 0.05). After 10 days of feeding the basic diet, FF concentrations in the muscle tissue of both groups decreased to comparable levels.

### 3.2. Histopathological Changes of the Shrimp Intestine

**Figure 1:**
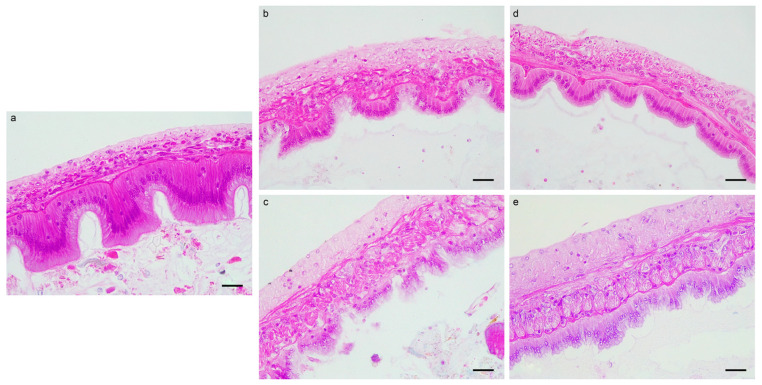
Intestinal histopathological observation of shrimp in control samples (**a**), D5 (**b**), T10 (**c**), D15 (**d**) and T20 (**e**). The black lines represents a scale bar, where 1 scale bar = 30 μm.

Histological examination of intestinal morphology revealed that the control samples exhibited well-preserved tissue structure integrity, characterized by an orderly connective tissue layer, tightly arranged epithelial cells, distinct intercellular spaces, and neatly organized, and intact microvilli (Figure 1a). In contrast, shrimp fed the FF diet for 5 days exhibited pronounced histopathological alterations (Figure 1b). The connective tissue layer appeared disorganized, cell boundaries were indistinct, and nuclei exhibited clustered. Epithelial cells displayed blurred boundaries, focal degeneration and necrosis, as well as nuclear pyknosis. Additionally, intestinal microvilli were significantly shortened and were nearly absent in certain regions. After 10 days of the FF exposure, shrimp in group T exhibited substantially more severe intestinal damage than those in group D (Figure 1c). The intestinal basal membrane was distorted and locally disrupted. Injured epithelial cells displayed extensive degeneration, necrosis, and detachment, accompanied by nuclear shrinkage and dissolution. Notably, the intestinal microvilli were completely absent.

Ten days after feeding the basic diet, partial recovery of intestinal damage was observed, although the tissue did not return to the pre-experimental baseline. In group D, the length of the intestinal microvilli was restored, and epithelial cells were clearly delineated and structurally intact; however, the epithelial layer remained thinner than that in the control group (Figure 1d). In contrast, group T showed delayed recovery of intestinal microvilli length, with epithelial cell boundaries remaining indistinct (Figure 1e). Epithelial cells continued to show degeneration and necrosis, accompanied by nuclear dissolution.

### 3.3. Changes in Microbial Community Diversity After FF Feeding

**Figure 2:**
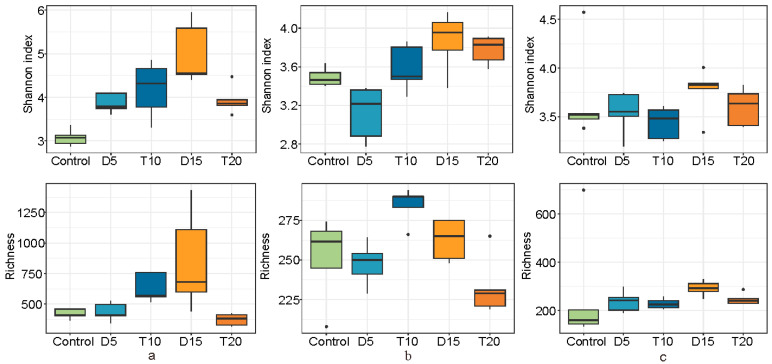
Dynamics of α-diversity indices of microbial community in rearing water (**a**), shrimp stomach (**b**) and intestine (**c**).

Compared with the control samples, the Shannon index of the rearing water increased significantly following FF feeding, with no significant differences between groups D and T (Figure 2a). In group D, the Shannon index of the shrimp stomach decreased significantly, while richness significantly increased in group T, showing a notable difference between the two groups (Figure 2b). However, no significant changes were observed in the α-diversity indices of the shrimp intestine (Figure 2c). After 10 days of feeding the basic diet, all α-diversity indices in group D exhibited a sharp increase, whereas those in group T showed an overall declining trend.

**Figure 3:**
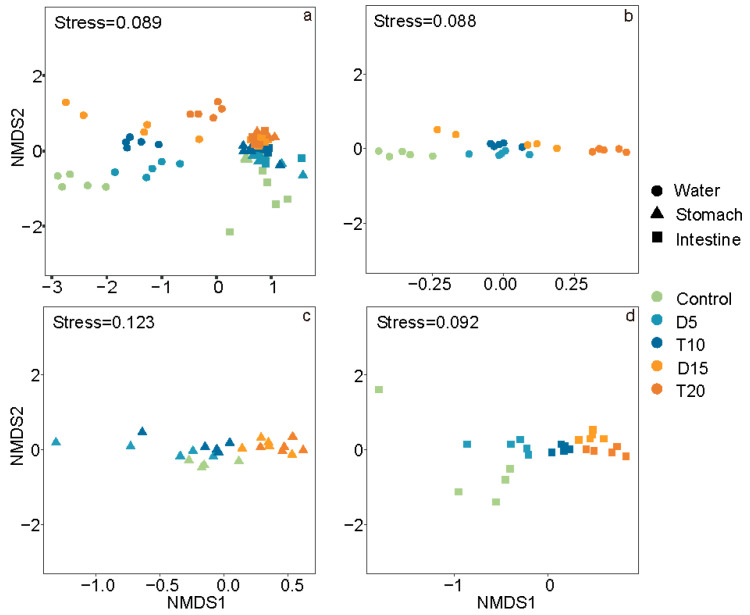
NMDS analysis of microbial community structure of total samples (**a**), rearing water (**b**), shrimp stomach (**c**) and intestine (**d**) based on the Bray–Curtis distance.

NMDS analysis revealed that the microbial communities of rearing water, shrimp stomach and intestine followed similar succession patterns and were distinctly separated along the NMDS1 axis (Figure 3). After FF feeding, microbial communities in groups D and T were generally more tightly clustered than those in control samples. ANOSIM analysis indicated significant differences in microbial communities between the FF-fed groups and the control samples, as well as between groups D and T at each sampling time, except for the comparison between D15 and T20 in the shrimp stomach (Table 3).

### 3.4. Microbial Community Compositions in Rearing Water, Shrimp Stomach and Intestine

**Figure 4:**
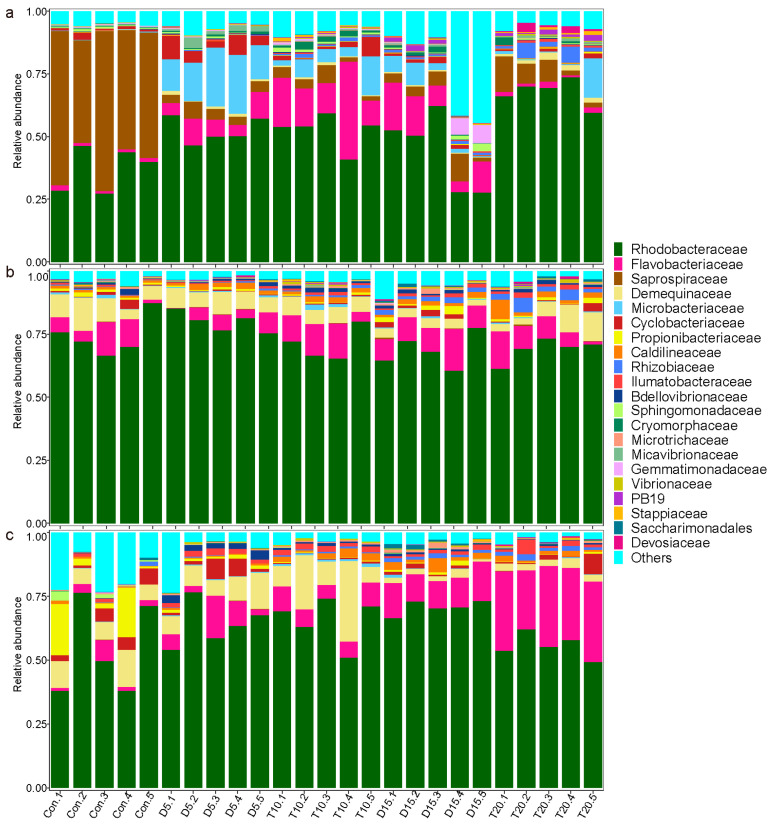
Relative abundance of microbial community in rearing water (**a**), shrimp stomach (**b**) and intestine (**c**) at family level.

In the control samples, microbial communities in the rearing water were predominantly composed of *Saprospiraceae* (52.86%) and *Rhodobacteraceae* (36.98%) (Figure 4a). In the shrimp stomach, the dominant families included *Rhodobacteraceae* (70.96%), *Demequinaceae* (8.87%) and *Flavobacteriaceae* (8.58%) (Figure 4b). Meanwhile, the shrimp intestine was primarily made up of *Rhodobacteraceae* (54.55%), *Propionibacteriaceae* (9.00%), *Demequinaceae* (8.82%), *Cyclobacteriaceae* (3.98%) and *Flavobacteriaceae* (3.31%) (Figure 4c). The microbial community compositions of the rearing water and shrimp intestine exhibited significant changes both during and after FF feeding, while only minor changes were observed in the stomach. After FF feeding, the relative abundances of *Rhodobacteraceae* and *Flavobacteriaceae* in both rearing water and intestine were generally enriched in groups D and T, with distinct trends emerging between the two groups after feeding with the basic diet. The relative abundance of *Saprospiraceae* in rearing water and *Propionibacteriaceae* in the shrimp intestine sharply decreased and did not recover during the subsequent experiment period. Additionally, *Microbacteriaceae* in the rearing water and *Demequinaceae* in the intestine increased significantly after FF feeding but declined after feeding with the basic diet. Furthermore, *Vibrionaceae* in all three habitats of groups D and T increased significantly after FF feeding, with consistently higher relative abundances observed in group T than in group D.

**Figure 5:**
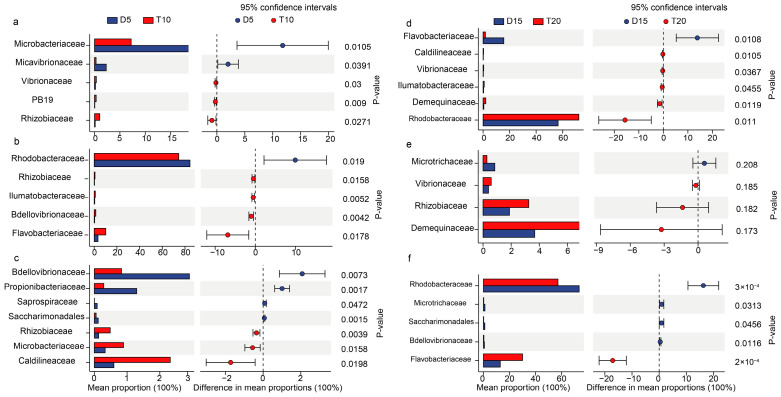
Differences in microbial community composition in rearing water (**a**,**d**), shrimp stomach (**b**,**e**) and intestine (**c**,**f**) between groups analyzed by STAMP method.

Further STAMP analysis revealed differences in microbial community composition between groups across the FF feeding and basic diet feeding phases (Figure 5). After FF feeding, the relative abundance of *Microbacteriaceae* was significantly higher in group D than in group T in rearing water but was significantly lower in the shrimp intestine. In the shrimp stomach, the relative abundance of *Rhodobacteraceae* was significantly higher in group D than in group T. In the shrimp intestine, the abundance of *Vibrionaceae* and *Flavobacteriaceae* was significantly higher in group T than that in group D. After feeding with the basal diet, the abundance of *Rhodobacteraceae* in the shrimp intestine was significantly higher in group D than in group T, whereas the abundance of *Flavobacteriaceae* was significantly lower in group D.

**Figure 6:**
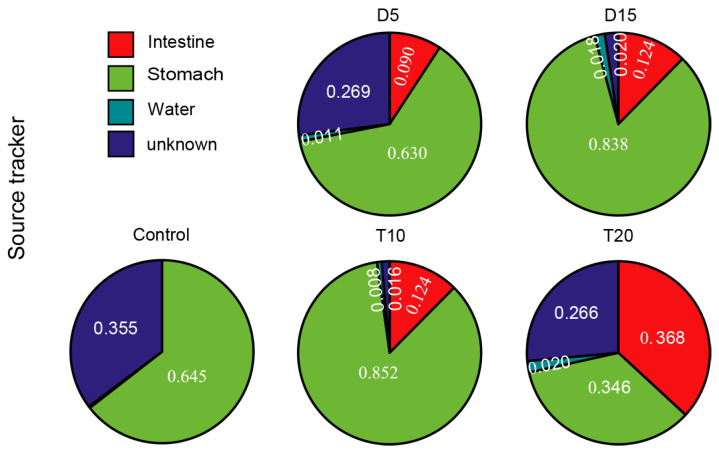
Proportions of the shrimp intestinal microbiota derived from rearing water and stomach microbiota.

To track the sources of microbes in the intestine, SourceTracker was employed to estimate the contributions of rearing water and stomach microbiota to the shrimp intestine (Figure 6). In the control samples, the proportions of intestine bacteria derived from rearing water and stomach microbiota were 0.003 and 0.645, respectively. After FF feeding, the proportions of ASVs in the shrimp intestine of group D originating from the control intestine, rearing water, and stomach microbiota were 0.090, 0.011, and 0.630, respectively, whereas in group T, these proportions were 0.124, 0.008, and 0.852. After 10 days of feeding with the basic diet, the contributions of the previous intestine, rearing water, and stomach microbiota to the intestinal bacterial community in group D 0.124, 0.018, and 0.838, respectively, compared with 0.368, 0.020, and 0.346 in group T.

### 3.5. Compositions of Bacterial Pathogens in Rearing Water, Shrimp Stomach and Intestine

**Figure 7:**
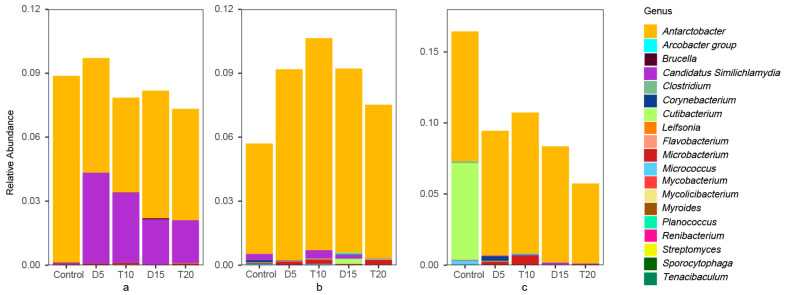
Relative abundance of pathogenic bacteria of rearing water (**a**), shrimp stomach (**b**) and intestine (**c**).

A total of 74, 80 and 94 ASVs corresponding to potentially pathogenic bacteria were identified in rearing water, shrimp stomach and intestine, representing 10, 13 and 18 genera, respectively (Figure 7). The relative abundance of bacterial pathogens was significantly higher in the intestine (3.06%) than in the rearing water (2.63%) and stomach (2.36%). Notably, *Antarctobacter* was the dominant pathogen across all three habitats, with relative abundances of 2.00%, 2.24%, and 2.56%, respectively.

After FF feeding, the relative abundance of bacterial pathogens increased significantly in the stomach, while it decreased significantly in the intestine. At the genus level, the relative abundance of *Candidatus similichlamydia* in the rearing water increased significantly, whereas the abundances of *Cutibacterium* and *Micrococcus* in the intestine decreased. Following 10 days of feeding with the basic diet, the relative abundances of bacterial pathogens in all three habitats decreased.

### 3.6. ARGs in Rearing Water and Shrimp Intestine

**Figure 8:**
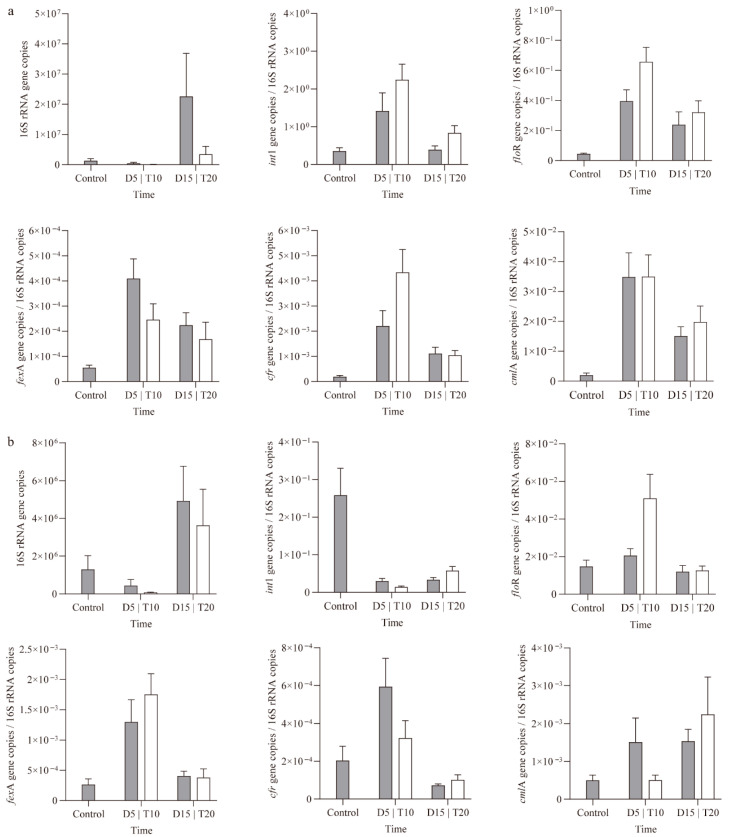
Changes in the abundances of ARGs in rearing water (**a**) and shrimp intestine (**b**).

Real-time qPCR results revealed the number of 16S rRNA gene copies in both rearing water and shrimp intestine decreased significantly after FF feeding but increased following 10 days of feeding with the basic diet (Figure 8). In the control samples, the concentrations of ARGs ranged from 5.56 × 10^−5^ copies/16S rRNA copies (*fex*A) to 4.19 × 10^−1^ copies/16S rRNA copies (*int*1) in rearing water, and from 2.04 × 10^−4^ copies/16S rRNA copies (*cfr*) to 2.58 × 10^−1^ copies/16S rRNA copies (*int*1) in the intestine.

After FF feeding, the abundance of target ARGs increased significantly in both rearing water and intestine for groups D and T, except for the *int1* gene in the intestine. In rearing water, group T exhibited higher abundances of *int1*, *floR*, and *cfr*, while *fexA* was more abundant in group D. In the intestine, group D showed higher levels of *int1*, *cfr*, and *cmlA*, whereas group T had higher levels of *floR* and *fexA*.

Following 10 days of feeding with the basic diet, the abundance of most ARGs declined across all habitats, except for *int*1 and *cml*A in the intestine. In rearing water, group T maintained higher abundances of *int*1, *flo*R, *fex*A, and *cml*A compared to group D. However, no significant differences were observed in intestinal ARG abundances between groups T and D.

### 3.7. Correlations Among ARGs, Microbial Communities and Bacterial Pathogens

**Figure 9:**
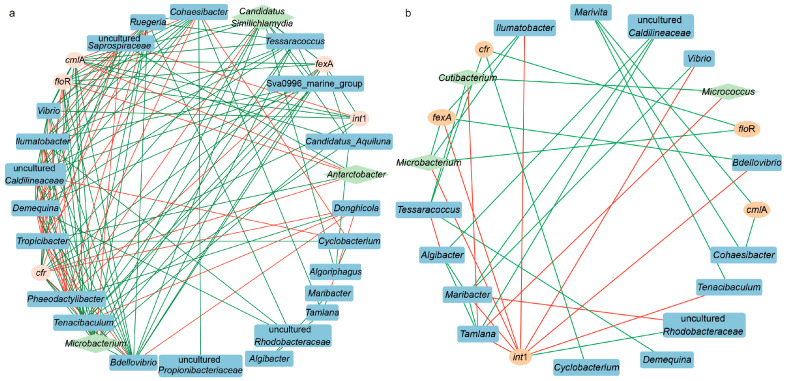
Co-occurrence networks illustrate interactions among ARGs, microbial communities, and bacterial pathogens in rearing water (**a**) and shrimp intestine (**b**). The blue, green, and orange nodes represent different bacterial genera, pathogenic bacterial genera and ARG types, respectively. Negative and positive correlations are indicated by red and green edges, respectively, and only significant correlations (*p* < 0.05) are shown.

Network analysis was employed to elucidate the co-occurrence patterns among ARGs, microbial communities, and bacterial pathogens. The co-occurrence network in rearing water comprised 30 nodes and 147 edges (Figure 9a), whereas the network in the shrimp intestine contained 24 nodes and 36 edges (Figure 9b). In the rearing water, several bacterial genera, together with the *int*1 gene, were associated with multiple ARGs. For instance, *Bdellovibrio*, *Tenacibaculum*, and *Vibrio* were positively correlated with *fexA*, *cmlA*, *floR*, and *cfr*, with *Vibrio* additionally showing a positive correlation with *int*1. *Candidatus aquiluna* was positively correlated with *fex*A and *cml*A, whereas the pathogenic bacterium *Microbacterium* correlated positively with *int*1, *cml*A, *flo*R and *cfr*. Similarly, *Candidatus similichlamydia* exhibited positive associations with *fex*A, *cml*A, *flo*R and *cfr*. In contrast, *Antarctobater* was negatively correlated with *flo*R, *cml*A and *cfr*. The *int*1 gene in rearing water showed a positive correlation with *fex*A, *cml*A, *flo*R and *cfr*. In the shrimp intestine, *Bdellovibrio* and *Ilumatobacter* were positively correlated with *fex*A, but negatively with *int*1, while *Tessaracoccus* and *Cyclobacterium* were positively correlated with *cfr*. *Vibro* showed a negative correlation with *int*1, and *Microbacterium* was positively associated with *flo*R and *fex*A but negatively with *int*1. The *int*1 gene in the intestine was positively correlated only with *fex*A. Collectively, these associations suggest that the aforementioned bacteria may serve as potential hosts for ARGs.

## 4. Discussion

Antibiotics commonly used in aquaculture, such as FF, are primarily derived from veterinary drugs, and are often selected by aquaculture producers based on empirical experience, which may lead to drug abuse in the absence of proper management. Although FF has been used as a feed additive and been considered a growth promoter in aquaculture [44], its relevance to growth promotion remains unclear. Moreover, some studies have reported toxicity and adverse side effects associated with excessive use of FF [7]. Therefore, there is an urgent need to elucidate how the effects of FF in shrimp are influenced by the duration of administration. This study addresses this gap by systematically investigating the administration duration of FF in shrimp farming, aiming to clarify the relationship between the duration and its corresponding effects. The findings are expected to provide evidence-based guidance for farming practices, thereby supporting the transition toward more scientific and precise use of antibiotics in aquaculture.

Short-term exposure to FF has been reported to promote the growth of flounder larvae (*Paralichthys olivaceus*), whereas prolonged exposure inhibits growth [45]. Our results are consistent with this observation, indicating that extended FF feeding duration can reduce the growth performance of shrimp. Similarly, a daily dose of 10 mg/kg body weight of FF for 10 days was shown to negatively impact the growth of *Dicentrarchus labrax* [46]. In contrast, other studies have reported opposing findings, noting that while repeated FF administration at doses of 15 and 30 mg/kg for 15 days slightly improved weight gain rate and specific growth rate, the effects are limited and not sufficient to consider its use as a growth-promoting strategy [47].

Regarding intestinal structure, FF exposure has been shown to significantly alter the intestinal villus architecture in *E*. *sinensis*, resulting in apical epithelial shedding and shortened villi [12]. In the present study, we similarly observed intestinal damage in shrimp, including blurred epithelial cell structure, necrosis, and markedly shortened or absent microvilli. These structural changes directly affect intestinal function, as the density of mucosal folds, microvilli thickness and the number of goblet cells are closely related to digestive and absorptive capacity [48]. Microvilli enhance the intestinal absorptive surface area, while goblet cells play an important role in host defense against microbial attachment and invasion [49]. Excessive antibiotic exposure can lead to the overaccumulation of endogenous reactive oxygen species (ROS) in aquatic organisms [50,51]. When ROS levels exceed the antioxidant capacity of tissues, oxidative defense homeostasis is disrupted, leading to severe cellular damage and increased tissue toxicity [7]. This oxidative stress mechanism may be a key factor contributing to antibiotic-induced disruption of intestinal structure and lead to functional impairment. Excessive antibiotic use can disrupt intestinal structure in aquatic animals, leading to intestine dysfunction. In our study, the severity of tissue injury increased with the duration of FF exposure, consistent with findings from study on sulfadiazine in *C*. *Auratus* [11]. Although intestinal damage showed partial recovery 10 days after stopping FF feeding, structural injuries persisted until the end of the experiment. This indicates that while FF adversely affects the shrimp’s intestinal structure during the feeding process, the intestine appears to gradually recover once FF administration is discontinued.

Bacterial community diversity in both the environment and the host is essential for host health and welfare [52]. Therefore, analyzing changes in the associated microbiome is of great significance for understanding the impact of FF feeding in shrimp. In this study, the α-diversity across the three habitats did not show consistent significant changes following FF feeding, a result that differs from findings from a previous study on channel catfish exposed to FF [19]. We hypothesize that the response of microbial α-diversity to FF is likely closely correlated with the host, as suggested in previous research [33]. Nevertheless, both NMDS and ANOSIM analysis revealed statistically significant differences in microbial β-diversity, indicating that the microbial community structure was altered during and after FF feeding [32,33].

In this study, *Saprospiraceae* and *Rhodobacteraceae* were the predominant families in the rearing water, while *Rhodobacteraceae*, *Demequinaceae* and *Flavobacteriaceae* were dominant in the shrimp stomach and intestine. The dominant bacterial composition of the shrimp-associated microbiome is consistent with findings from previous studies [53,54]. Significant shifts in bacterial composition were observed after FF feeding, particularly in the rearing water and shrimp intestine. *Rhodobacteraceae* consistently dominated across all three habitats throughout the experimental period, with an increasing trend following FF feeding. Notably, ten days after feeding the basic diet, the relative abundance of *Rhodobacteraceae* in the intestine of group D was significantly higher than that in group T. Many studies have reported that a high relative abundance of *Rhodobacteraceae* in the intestine exerts positively correlated with shrimp health, growth and disease resistance [55,56,57]. In contrast, the relative abundance of *Flavobacteriaceae* in the intestine was significantly lower in group D compared to group T, and a reduced abundance of this family is generally associated with beneficial health phenotypes in shrimp aquaculture [58]. Members of the *Flavobacteriaceae* family, particularly belonging to the genera *Tenacibaculum* and *Flavobacterium*, are known as opportunistic pathogens in marine and freshwater fishes [59,60]. A previous work has shown that *Flavobacteriaceae* species can suppress candidate pathogens in healthy shrimp, although this relationship may shift to a synergistic effect in diseased shrimp [61]. Antibiotic treatment can disrupt the gut microbiota by eliminating key microbial populations essential for maintaining intestinal function. The resulting decline in microbial diversity creates ecological niches that can be exploited by opportunistic pathogens, which often carry transferable ARGs or possess adaptive traits such as spore formation, enabling them to survive and proliferate under antibiotic pressure [62]. SourceTracker analysis implied that the microbiota in the shrimp intestine primarily originated from stomach microbes rather than from rearing water, supporting earlier findings that the bacterial composition shows little similarity between water and shrimp intestine [21,63]. Furthermore, we also found that the proportion of intestinal microbes sourced from the stomach significantly increased with extended FF feeding duration. The perturbation in the intestinal microbial community due to prolonged FF feeding significantly influenced the composition of the subsequent intestinal microbial community.

In general, antibiotics are an optional choice for preventing or treating bacterial diseases by inhibiting pathogenic bacteria [27]. In the present study, only the relative abundance of potential pathogens in the shrimp intestine decreased sharply following FF feeding. At the genus level, *Antarctobacter* in the rearing water and *Cutibacterium* in the intestine showed a decreasing trend with FF intervention. *Antarctobacter* was classified as a fish bacterial pathogen based on genomic data in the KEGG database [64], although other study suggests that *Antarctobacter* sp. belongs to the *Roseobacter* clade, which suppresses the growth of the fish pathogen *Vibrio anguillarum* [65]. *Cutibacterium* belongs to the cutaneous group of bacteria in humans and has been implicated in the pathogenesis of acne vulgaris [66]. In contrast, the abundance of *Candidatus similichlamydia* in the rearing water increased after FF feeding and continued to maintain a high level of abundance at the end of the experiment. Members of *Candidatus similichlamydia* were reported to be associated with epitheliocystis in the gills and skin epithelia of fish such as *Salmo trutta* and *Epinephelus coioides* [67,68].

In this study, a significant enrichment of target ARGs was observed in both the rearing water and shrimp intestine following FF feeding, confirming that oral antibiotic administration can induce ARGs accumulation [69]. The abundance of ARGs in rearing water increased with extended FF feeding duration, while the trends for different ARGs in the shrimp intestine varied inconsistently. The *Int*1 gene showed the highest abundance in both rearing water and intestine samples and has been reported to drive the horizontal transfer of ARGs among bacterial communities, thereby contributing to the emergence of multidrug-resistant bacteria [70]. Since ARGs are part of bacterial genomes and primarily disseminated via mobile genetic elements, changes in the abundance of ARGs are closely associated with shifts in bacterial populations [71]. Previous studies also detected the co-occurrence of various mobile genetic elements (MGEs) with antibiotic resistance genes (ARGs) following FF administration [29]. According to co-occurrence network analysis, several bacterial genera, including probiotic and potential pathogenic bacteria, were significantly positively correlated with multiple ARGs. Given that the *int*1 gene exhibited stronger correlations with bacterial genera in the rearing water, compared to the shrimp intestine, we speculated that the rearing water may serve as a reservoir for ARGs dissemination [72]. However, due to the absence of real-time monitoring of FF concentrations and physicochemical parameters in the rearing water, the responses of ARGs and MGEs to FF exposure remain insufficiently understood and require further investigation. Future research could adopt integrated multi-omics approaches, such as the combination of transcriptomics and metabolomics, to systematically elucidate the gene regulatory networks and metabolic response mechanisms of intestinal microbiota in shrimp under FF exposure, thereby providing a theoretical foundation for accurately assessing the antibiotic’s ecotoxicological effects. Furthermore, research should aim to clarify the causal relationship between FF-induced microbiota shifts and disease outbreaks in shrimp. Building on these insights, subsequent investigations could explore strategies—such as applying beneficial substances—to eliminate or mitigate the adverse effects of FF on the microbial community.

## 5. Conclusions

In summary, extending the duration of FF feeding significantly increased FF concentration in shrimp muscle and slightly decreased shrimp growth. The intestinal damage caused by FF feeding was aggravated with extended duration and gradually recovered after FF withdrawal. FF feeding also led to significant shifts in microbial composition and β-diversity in both rearing water and shrimp intestine. In particular, extended FF feeding duration significantly influenced on the subsequent composition of the intestinal microbial community and also induced lower abundance of *Rhodobacteraceae* along with higher abundance of *Flavobacteriaceae* and *Vibrionaceae* in the intestine after 10 days of feeding the basic diet, which may be associated with the progression of shrimp disease. Based on correlation analysis of ARGs, microbial communities and pathogenic bacteria, we speculated that the rearing water may serve as a reservoir for ARGs dissemination compared to the shrimp intestine. These findings provide valuable insights for the duration of FF administration in shrimp farming and highlight the potential risks associated with the excessive use of FF.

## Figures and Tables

**Table 1:** qPCR primers for ARGs, integron and the 16S rRNA gene.

Genes	Sequences (5′~3′)	Annealing Temp (°C)	Amplicon Size (bp)	Ref.
16S rRNA	FW GGTAGTCYAYGCMSTAAACG RV GACARCCATGCASCACCTG	62	263	[37]
*int*1	FW GGCTTCGTGATCICCTGCTT RV CATTCCTGGCCGTGGTTCT	62	196	[38]
*flo*R	FW CGGTCGGTATTGTCTTCACG RV TCACGGGCCACGCTGTAT	56	171	[39]
*cml*A	FW GCCAGCAGTGCCGTTTAT RV GGCCACCTCCCAGTAGAA	55	158	
*cfr*	FW TGTGCTACAGGCAACATTGGAT RV CAAATACTTGACGGTTGGCTGAG	55	148	
*fexA*	FW ATTCTCCCGCAAATAACG RV TCGGCTCAGTAGCATCACG	52	156	

**Table 2:** Growth performance of shrimp and the residual of FF.

Index	Group D	Group T	*p*
Survival rate (%)	86.67 ± 4.86	81.00 ± 5.96	0.14
Specific growth rate (%/d)	4.19 ± 0.69	3.67 ± 0.86	0.32
Length growth rate (%/d)	1.78 ± 0.44	1.58 ± 0.40	0.46
FF concentration in muscle after FF feeding (ug/g)	1.07 ± 0.02	1.38 ± 0.04	0.02
FF concentration in muscle after 10 days of feeding basic diet (ug/g)	0.048 ± 0.001	0.041 ± 0.004	0.42

**Table 3:** Analysis of similarity (ANOSIM) based on Bray–Curtis distance.

Sample	ANOSIM	*R*	*p*	ANOSIM	*R*	*p*
Rearing water	Control/D5	1	0.009	D5/T10	0.696	0.008
	Control/T10	1	0.01	D15/T20	0.548	0.013
Stomach	Control/D5	0.95	0.008	D5/T10	0.552	0.009
	Control/T10	1	0.008	D15/T20	0.356	0.054
Intestine	Control/D5	0.51	0.012	D5/T10	0.66	0.016
	Control/T10	0.704	0.006	D15/T20	0.828	0.017

Notes: an *R* value close to 1 indicates that the difference between groups is greater than that within the group.

## Data Availability

The raw sequence data reported in this paper have been deposited in the Genome Sequence Archive (Genomics, Proteomics & Bioinformatics 2021) in National Genomics Data Center (Nucleic Acids Res 2022), China National Center for Bioinformation/Beijing Institute of Genomics, Chinese Academy of Sciences (GSA: CRA019177) that are publicly accessible at https://ngdc.cncb.ac.cn/gsa, accessed on 23 September 2024.
